# Musculoskeletal complaints, physical work demands, and functional capacity in individuals with a brachial plexus injury: An exploratory study

**DOI:** 10.3233/WOR-220680

**Published:** 2024-03-08

**Authors:** Tallie M.J. van der Laan, Sietke G. Postema, Siawash A. Alkozai, Corry K. van der Sluis, Michiel F. Reneman

**Affiliations:** Department of Rehabilitation Medicine, University of Groningen, University Medical Center Groningen, Groningen, The Netherlands

**Keywords:** Work capacity evaluation, pain, upper extremity, work performance, workload, ergonomic assessments

## Abstract

**BACKGROUND:**

Musculoskeletal complaints (MSCs) may be more common in individuals with brachial plexus injury (BPI), whose physical work demands exceed their functional capacity (FC).

**OBJECTIVES:**

(a) To assess the concurrent validity of five methods for measuring upper extremity work demands and the Dictionary of Occupational Titles (DOT). (b) To explore the relations between MSCs, physical work demands, and FC in individuals with BPI.

**METHODS:**

This study had a descriptive correlational design. Physical work demands of 16 individuals with BPI (12 males, 6 one-handed workers) were assessed during work using five assessment methods and the DOT. Spearman correlation coefficients between work demand methods were determined. FC was assessed using the functional capacity evaluation one-handed (FCE-OH). A questionnaire was used to examine MSCs. The relationship between MSCs, physical work demands and FC was analyzed visually, using Spearman correlation coefficients, and by comparing FCE-OH results to FCE reference values.

**RESULTS:**

Spearman correlation coefficients for the DOT and four out of five assessment methods for determining work demands on upper extremities were significant and moderate (four combinations: *r* = 0.65–0.79) to strong (five combinations: *r* = 0.81–0.94). Correlations of the fifth method with the other methods were weak to fair. No significant relationships were found between MSCs, physical work demands and FCE-OH results.

**CONCLUSION:**

The relationships between MSCs, physical work demands, and FC are evidently complex and require further investigation. In this small sample the concurrent validity of the DOT and four methods for determining work demands on upper extremities was moderate to good.

## 1 Introduction

Individuals with a brachial plexus injury (BPI) have functional deficits in the affected upper limb. The nature of these deficits varies, depending on which nerve roots and peripheral nerves are impacted and the degree of damage. Most who have sustained BPI are young and have many working years ahead [1]. Fifty to seventy-nine percent of this population experience neuropathic pain as a result of the peripheral nerve lesions. In addition, almost half report musculoskeletal complaints (MSCs) in the non-affected bodily structures [2–4]. MSCs are defined as complaints in the muscles or joints that are not caused by trauma or a systemic disease [5]. Compared to the general population, those with BPI more frequently describe pain and stiffness of the neck, upper back and unaffected shoulder [4].

MSCs, as a secondary consequence of BPI, can be aggravated by work demands [5]. Risk factors for work-related MSCs are known to be persistent static muscle contractions, awkward postures, forceful exertions, and repetitive movements [6, 7]. These risk factors are of special significance for individuals with BPI because of the need for the worker to develop compensatory strategies that involve greater use of unaffected bodily structures [8]. Given similar work demands, the physical stresses on the unaffected limb and adjacent structures in a person with BPI are likely to be greater, compared to individuals with unimpaired two-handed function. Therefore, workers with BPI may be at greater risk to develop MSCs because of a possible mismatch between physical work demands and their functional capacity.

Work-related MSCs may be prevented by matching physical work demands to a person’s functional capacity, which is defined as “the highest probable level of functioning that a person can achieve in a given domain at a given moment within a standardized environment considering multiple biopsychosocial factors including personal and environmental factors” [9]. In order to minimize or prevent MSCs in workers with BPI, clear understanding of physical work demands and whether they are matched to the individual’s functional capacity, is therefore imperative. This calls for valid approaches to assessing both the physical work demands and the individual’s functional capacity, which can then lead to strategies for adjusting physical work demands in a way that decreases the likelihood of MSCs. Available tools are the Dictionary of Occupational Titles (DOT) database, functional capacity evaluations and observational ergonomic assessment tools assessing physical work demands.

A well-established source of information on physical work demands is the Dictionary of Occupational Titles (DOT), which offers a set of widely used standardized occupational descriptions. This information serves as a valuable tool to support job placement, and it is available via an online database called the Occupational Information Network [10]. Descriptions include specific task elements, some of which may require upper extremity functions such as gripping, dexterity, applying torque, lifting, pushing and pulling. Occupations in the DOT are grouped into five categories –sedentary, light, medium, heavy, and very heavy according to the bodily strength required for their performance [10]. It should be noted that this classification system appears to lack validity as a means for guiding vocational decisions for individuals with hand and upper extremity limitations [11].

Functional capacity evaluations (FCE) are instruments used to evaluate an individual’s capacity to perform activities so that recommendations can be made for their work participation, while taking account of their body structure and functions, environmental and personal factors, and health status [9]. The functional capacity of individuals with BPI can be determined using the functional capacity evaluation one-handed (FCE-OH), which is a short form capacity evaluation for persons with only one functional hand or one hand with a limited functionality [12]. FCE reference values have been established for each DOT category, covering the working population [13], whether or not these reference values can be applied to individuals with BPI remains unclear because the matching of DOT categories to the physical job demands of individuals with BPI has not been established.

We identified four observational ergonomic assessment tools, that have merit for measurement of the exposure of workers to risk factors associated with MSCs of the upper extremity. These are the Rapid Upper Limb Assessment (RULA) [14], Strain Index (SI) [15], Occupational Repetitive Actions (OCRA) [16] and the Threshold Limit Value for Hand Activity Level (TLV for HAL) [17]. Additionally, the revised six-item Upper Extremity Work Demands questionnaire (UEWD-R) was determined to be a useful means to assess perceptions of upper extremity work demands [18]. In previous studies conducted with general populations, the investigators found that high risk scores on the RULA, TLV for HAL, and SI, were associated with an increased risk of MSCs. For example, neck, back and wrist complaints were associated with high RULA scores (neck complaints odds ratio 2.1, *p* = 0.02; back complaints *p* = 0.02; wrist complaints *p* < 0.01)) [19, 20]. High scores on the TLV for HAL were associated with carpal tunnel syndrome (odds ratio 1.48, hazard ratio 2.01) [21–23]. Furthermore, high SI scores were associated with trigger fingers (hazard ratio 3.1) and carpal tunnel syndrome (odds ratio 1-48–1.66, hazard ratio 2.1) [21–23]. In the general population, none of these methods was preferred above the others [24]. We anticipated that these methods for measuring physical work demands would also be applicable to the BPI population.

The primary aim of this study was to apply existing systems to describe physical work demands of individuals with BPI and to improve the knowledge on how these physical work demands are related to functional capacity and the presence of MSCs in individuals with BPI. Knowledge on the influence of physical factors in the development of MSCs may help to take preventive measures that may reduce the risk of MSC in this population. The first part of this study focuses on methods that assess physical work demands of the upper extremity. The aim of this part was to determine the concurrent validity, that is, the extent of agreement of five methods for measuring upper extremity work demands, one self-reported questionnaire and four observation-based methods and the DOT categorization of individuals with BPI. We hypothesized that strong correlations would be found between the five methods for measuring upper extremity work demands, because all the methods measure similar constructs, even though there are some differences in operational definitions. We further hypothesized that correlations between the five upper extremity work demands methods and the DOT categorization would be moderate, because the DOT was not specifically developed to assess upper extremity work demands and only considers some aspects of the observation methods. In the second part we aim to explore the relationship among MSCs, physical work demands and functional capacity in individuals with BPI. We hypothesized that MSCs are more common in individuals with BPI whose physical work demands exceed their functional capacity, reflecting a mismatch between work demands and functional capacity.

## 2 Methods

### 2.1 Design

This study has a descriptive correlational research design, and is comprised two parts. In the first part we assessed physical work demands, while in the second part we explored the degree of relationship among the presence of MSCs, the physical work demand assessments, and the results of the functional capacity evaluation for one-handed individuals (FCE-OH) tests.

### 2.2 Participants

Between February 2016 and July 2017 and between October 2018 and May 2019, we recruited 23 individuals with unilateral BPI (19 males, 4 females, mean age 49.7±10.4 years), all of whom had participated in a previous study aimed at determining their functional capacity, and asked for permission to observe them at their workplaces to evaluate their upper extremity work demands [25]. There had been a pause in recruitment because of unforeseen absence of the main researcher. The inclusion criteria of the previous study were the same for the current study. All participants were aged between 18 and 65 years, performed paid work, had an adequate understanding of the Dutch language, had normal function of the unaffected hand, and did not have any conditions that could harm their safety during physical effort, such as hypertension (blood pressure >160/100 mmHg at rest) or serious pulmonary and/or cardiac conditions. The physical activity readiness questionnaire (PAR-q) was used to screen participants for these conditions [26]. Every participant provided written informed consent before the work observation took place. The local medical ethics committee decided to waive formal approval (METC file number: METc 2016/508). All procedures were followed in accordance with the 1975 Declaration of Helsinki, as revised in 2000.

### 2.3 Physical examination

During the previous study all participants underwent a physical examination to determine remaining activity on the affected side and to check if the unaffected side retained normal function [25]. Their active range of motion was assessed, and the strength of hand and wrist muscles was determined using the Medical Research Council (MRC) scale, which has good concurrent validity (Spearman’s rho *(r)* = *0.78)* and satisfactory inter-rater and intra-rater reliability (kappa values 0.78–0.88) [27]. Appendix 1 provides a summary of the assessments of tested muscles. Sensation-threshold detection was assessed using Semmes-Weinstein monofilaments. The palmar side of the thumb, index finger, and little finger were each touched three times with the monofilament 2.83. If the participant sensed two out of three touches, their threshold detection was considered normal. The test was repeated with monofilament 3.61 (the threshold for diminished touch) and monofilament 4.31 (the threshold for diminished protective sensation) if fewer than two out three touches were sensed [28]. The intra-rater reliability was good (kappa values 0.80–0.89) and inter-rater reliability was satisfactory to good (kappa values 0.75–0.79; intraclass correlation coefficient 0.97). The concurrent validity was satisfactory to good (*r* between 0.57–0.65)[29, 30].

### 2.4 Musculoskeletal complaints

The “Health 2” component of the Dutch Musculoskeletal Questionnaire (DMQ) was used to assess MSCs. It contains 11 items for participants to rate their pain in each body part using an eleven-point numeric rating scale (0 = no pain to 10 = extreme pain) [31]. Participants were instructed to rate pain in their muscles and joints that was not caused by a trauma or systematic disease [5]. The DMQ was developed to assess musculoskeletal work load and work-related risk factors on MSCs and shows satisfactory agreement with physical examination for identifying low back pain (kappa 0.56–0.78) [31]. The “Health 2” component of the DMQ does not differentiate between MSCs and neuropathic pain. Only complaints meeting the definition of an MSC in body parts other than the affected side were classified as such.

### 2.5 Measurements for part 1

#### 2.5.1 Questionnaire

Prior to the commencement of work observation, participants were asked to fill out a questionnaire based on the OCRA checklist (see below) and the six-item questionnaire UEWD-R. The UEWD-R can be used to screen work demands on extremities and includes items addressing force, posture, and repetitive movements of the upper extremities during work. All questions were answered using a four-point Likert scale, with responses ranging from “rarely or never” to “almost always” [32]. Test-retest reliability (intraclass correlation coefficient 0.79) and construct validity (82% of the predefined correlations with the RULA were confirmed) of the UEWD-R in the general working population are reportedly good[18].

#### 2.5.2 Work observation

From March to July 2017 and from October 2018 to May 2019, we made video recordings at the participants’ workplaces while they performed their regular work tasks. They were filmed for 15 minutes from the frontal plane perspective and 15 minutes from the sagittal plane perspective. If similar actions continued for the next 30 minutes, the recording was terminated. However, if new work tasks were performed, the procedure was repeated and participants were filmed for another 30 minutes. Objects that were lifted during the tasks were weighed if possible; otherwise estimations were made, namely <2 kg, 2–10 kg, or more than >10 kg, in line with the RULA force score. As a final step, participants were asked whether the observed tasks matched their regular job-related activities (they answered “yes” or “no”) and, if applicable, how they diverged from these activities.

#### 2.5.3 Assessment of work observations: determination of physical work demands on the upper extremities

Work demands were rated by analyzing the videos recorded during work observations using the four scoring systems described in this section. All selected observation scoring systems were suitable for research purposes and measure workload on the upper extremity [24]. All observations were performed by one rater (SAA) in consultation with a second rater (TMJL).

The RULA method was designed to evaluate the risk factors for work-related MSCs in the upper limbs without the need for advanced ergonomic knowledge or expensive equipment [14]. It entails assigning four subscores. Subscore A evaluates the posture of the arms and wrists, and subscore B evaluates the postures of the neck, trunk, and legs. Subscore C is a combination of subscore A and muscle use and force involving the arms and wrists. Subscore D comprises a combination of subscore B and muscle use and force involving the neck, trunk, and legs. The combination of subscores C and D yields a RULA grand score of 1–7 [14]. Table 1 provides explanatory details for interpreting the grand score. Within the general working population, the intra-rater reliability is 91.7% and the inter-rater reliability is 94.6% [33, 34].

**Table 1:** Observational methods examining physical work demands of the upper extremity

Method	Measures physical work demands of	Range score	Interpretation score
RULA	Distal and proximal upper limb, wrist, neck, trunk and legs.Includes posture, force, repetitions and static postures of both the upper and lower extremities.	1 to 7	1-2: green, acceptable workload3-4: green, further investigation needed5-6: yellow, adjustments needed on short notice7: red, immediate improvement needed
OCRA checklist	Distal and proximal upper limbIncludes repetitiveness, force, posture and movements of the upper limb, recovery periods and additional factors (like cold, vibration, wearing gloves).	<7.5 to > 22.5	≤7.5: green, acceptable risk on MSC7.6–11.0: yellow, uncertain risk on MSC11.1–22.5: red, medium risk on MSC>22.5: purple, high risk on MSC
SI	Hand and wristIncludes: intensity, cycle duration, posture, efforts per minute, speed of work and duration of a task per day.	<3 to > 10	<3: green, probably safe3–10: yellow, increased risk for distal upper extremity disorder>10: red, probably hazardous.
Hand activity for TLV	Hand wrist and forearmIncludes: hand force and hand activity based on frequency, recovery time and speed of motion.	<0.56 to≥0.78	<0.56: green, below action limit0.56–0.77: yellow, slightly elevated risk on MSC≥0.78: red, significant elevated risk on MSC

*Abbreviations:* Hand activity for TLV, Hand activity for Threshold Limit Value; OCRA, Occupational Repetitive Actions; RULA, Rapid Upper Limb Assessment and SI, Strain Index.

The OCRA checklist (version 2013) is based on the OCRA index but is simpler in use. It is recommended to be used a screening tool for the presence of the main risk factors for biomechanical overload of the upper limbs is recommended [35]. The following risk factors were identified and qualified through observation: repetitiveness, force, awkward postures and movements, and lack of recovery periods. Additional factors, such as cold, vibration, glove use, and control over the work pace, were identified using a questionnaire (see Appendix 2) [35]. The OCRA checklist score was calculated for each upper limb using the following formula: (frequency+power+posture+additional factors)×recovery×duration. The scores ranged from < 7.5 to > 22.5. (Table 1) [35]. The intra-rater reliability (Cohen’s kappa 0.43) and inter-rater reliability (Fleiss kappa 0.52, intraclass correlation coefficient 0.80) of the OCRA checklist were moderate to good in the general working population [36].

The SI was developed to detect the jobs associated with distal upper-extremity disorders [15]. Six task variables (intensity of exertion, duration of exertion per cycle, effort per minute, wrist posture, speed of exertion, and duration of task per day) were assessed. Each task variable was given a value, known as a multiplier. The SI is the product of these six multipliers and is determined for both upper limbs separately [15]. Table 1 provides explanatory details for interpreting the score. Within the general working population, intra-rater reliability was moderate (intraclass correlation coefficient 0.59) and the test-retest reliability was moderate to good (intraclass correlation coefficient 0.52–0.82) [37, 38].

In 2018, the TLV for HAL was revised through adjustments made to the thresholds for determining the risk of overloading the hand, wrist, and forearm in repetitive hand movements and renamed the Hand activity TLV [39]. The revised method combines the hand activity and hand force variables. Hand activity, which is expressed on a visual analog scale ranging from 0 to 10, addresses exertion frequency, recovery time, and the speed of motion. Accordingly, 0 indicates no activity and 10 indicates the highest conceivable activity level. Hand force is estimated using a modified Borg CR10 scale for measuring perceived effort. The Hand activity TLV score was calculated by dividing the hand force Borg CR10 score by the hand activity score (see Table 1) [39]. Within the general working population, the inter-rater reliability of hand activity was good (intraclass correlation 0.71) and moderate for hand force (intraclass correlation 0.60) [40].

#### 2.5.4 Assessment of work observations: determination of DOT

Based on the video recordings and the measured weights lifted during the work observation, the occupation of the participants was categorized in a DOT category by two raters in consultation with each other. The categories are (1) sedentary work, (2) light work, (3) medium work, (4) heavy, and (5) very heavy work [14]. The categories were based on the maximal force and how frequently this force needed to be applied in order to perform the job.

#### 2.5.5 Analyses

The concurrent validity of the four work observation methods and the UEWD-R and DOT categories was determined using Spearman’s rank-order correlation coefficients. Correlation coefficients were determined for all combinations of physical work demand assessment methods. Correlations between risk levels assessed with the OCRA, SI, RULA, and Hand activity for TLV methods were also determined. To compare risk levels, the green and yellow risk levels of the OCRA and both green risk levels (scores 1–2 and 3–4) for the RULA were combined and categorized as low risk. The red score for the OCRA and the yellow scores for the other methods were categorized as medium risk, and the OCRA purple score and red scores for the other methods were categorized as high risk. Correlation was interpreted as strong when Spearman’s Rho (*r*) was≥0.8; moderate if 0.8 < r≥0.6; fair if 0.6 < r≥0.3; and weak if r was < 0.3 [41]. Correlation coefficients were considered significant if *p*-values were < 0.05. Concurrent validity was assumed satisfactory if r was≥0.8.

### 2.6 Measurements for part 2

#### 2.6.1 Functional capacity

Functional capacity was assessed using the FCE-OH [12], and was administered as a component of a previous study [25]. The FCE-OH is a short-form FCE for persons with only one functional hand or one functional hand and one hand with limited functionality. It comprises six FCE tests: two two-handed tests (the overhead lifting two-handed test and the overhead working test) and four one-handed tests (the overhead lifting one-handed test, the repetitive reaching test, the fingertip-dexterity test, and the hand-grip strength test). Participants performed the two-handed tests with two hands if possible; if this was not possible because of loss of function, the tests were performed using only the unaffected side. One-handed tests were performed with the affected and unaffected sides if possible. The objectives, test descriptions, and FCE outcomes for each test are described in Appendix 3.

#### 2.6.2 Physical work demands

The physical work demand methods assessed in part 1 of this study with a moderate to strong correlation (*r*≥0.6) were used to examine the relationship between physical work demands, functional capacity, and MSCs. If individuals performed more than one task during the work observation period, work demands were determined for each of these tasks. Because the FCE-OH tests measure the highest probable level of functioning a person can achieve, we selected the work tasks that required the highest physical work demands if multiple tasks were performed during the work observation. The work observations were performed following the implementation of the FCE-OH tests. Ideally, the time between implementation of the FCE-OH tests and work observation was maximally six weeks. However, if this was not possible, additional questions were asked to control for any changes in work and MSCs. The additional items focused on changes in work and work circumstances, with the addition of the DMQ “Health 2” part. Changes in work or working conditions or in scores recorded for the DMQ “Health 2” component that differed by more than three points relative to the first administration were deemed clinically relevant [42], necessitating repetition of the test.

#### 2.6.3 Analyses

Three methods were used to determine relations among MSCs, physical work demand assessments, and FCE-OH test results: (1) observation (visual), (2) determining Spearman’s Rho, and (3) use of FCE reference values [23]. Cases were omitted from all analyses if there were missing data for the MSC component of the questionnaire. Cases entailing missing data in the FCE tests or for one of the methods used to determine physical work demands were omitted from the analysis pertaining to the particular FCE-OH test or work observation method. Data analysis was performed using the SPSS statistical package for Windows (version 25.0; SPSS Inc., Chicago, IL, USA).

1. Visual assessment of scatter plots depicting all combinations of the overhead lifting test results (one- and two-handed) and physical work demand assessment methods was performed. In each plot, individuals with MSCs (MSC+) and without MSCs (MSC-) were labeled. The overhead lifting test results were chosen because these FCE tests were most commonly associated with return to work among individuals with complaints involving the upper extremities [43].
2. Spearman’s Rho was determined for all combinations of MSCs, physical work demand assessment methods, and FCE-OH test results. We anticipated no correlations or weak correlations between physical work demands and FCE test results because an individual does not necessarily need their entire available functional capacity to perform a job. For example, an individual with a sedentary job, who frequently plays sports, could demonstrate a high functional capacity.
3. FCE reference values established for the general working population were used to identify mismatches between functional capacity and physical work demands [13]. No FCE reference values were available for the one-handed overhead lifting test or the repetitive reaching test because of the required adjustments to these tests to make the tests suitable for one-handed individuals [12]. Therefore, mismatches could not be determined for these tests. Mismatches between FCE test results and DOT categories were determined separately for participants with and without MSCs. An FCE test result below the tenth percentile of the reference FCE value signified a mismatch [44]. Chi-square tests were applied to determine whether the number of matches differed between individuals with and without MSCs. Because of the exploratory nature of this study, a *p*-value of < 0.1 was considered significant. A Mann-Whitney U test was conducted to analyze differences between the MSC+and MSC- groups relating to physical work demands and FCE-OH test results.

## 3 Results

Of 23 eligible individuals, 16 participated in this study (Table 2). Seven individuals declined work observation because their employers did not agree (*n* = 2), they did not consent to being video-recorded at work (*n* = 2), or for an undisclosed reason (*n* = 3).

**Table 2:** Characteristics of the participants

	Individuals with BPI	MSC+	MSC-
	(*n* = 16)	(*n* = 9)	(*n* = 7)
Gender (male)	12 (75.0)	7 (77.8)	5 (71.4)
Age [median (IQR)]	54.0 (47.8–59.8)	49.0 (50.5–61.0)	50.0 (33.0–51.0)
Time since onset BPI in years [median (IQR)]	8.0 (2.5–38.3)	8.0 (1.5–37.5)	8.0 (4.0–50.0)
Cause of BPI
–Trauma	13 (81.3)	8 (88.9)	5 (71.4)
–BPBI	1 (6.3)	0 (0.0)	1 (14.3)
–Radiotherapy	1 (6.3)	0 (0.0)	1 (14.3)
–Unknown	1 (6.3)	1 (11.1)	0 (0.0)
Side (right)	8 (50.0)	5 (55.6)	3 (42.9)
Strength of tested muscles affected side (MRC scale 0–5)^∞^
–All tested muscles≥3	4 (25.0)	2 (22.2)	2 (28.6)
–One or more muscles < 3	10 (62.2)	6 (66.7)	4 (57.1)
–All tested muscles≤1	2 (12.5)	1 (11.1)	1 (14.3)
Threshold detection affected hand^#^
–Normal	0 (0)	0 (0.0)	0 (0.0)
–Diminished light touch	8 (25.0)	4 (44.4)	4 (57.1)
–Diminished protective sensation	4 (12.5)	2 (22.2)	2 (28.6)
–No threshold detected	4 (12.5)	3 (33.3)	1 (14.3)
Pain	11 (68.8)	9 (100.0)	2 (28.6)
Musculoskeletal complaints	9 (56.3)	9	NA
–1 location		6
–2 locations		2
–4 locations		1
Locations [*n*, median NRS (IQR)]
–Neck		5, 3.0 (2.0–5.0)
–Unaffected shoulder		3, 2.0 (2.0–3.0)
–Back		4, 3.0 (2.5–3.0)
–Hip		2, 4.5 (4.3–4.8)
–Foot		1, 4.0 (NA)
Performs work 1-handed	6 (37.5)	2 (22.2)	4 (57.1)
Profession (DOT^*^)
–Administrative work (1)	9 (56.3)	5 (55.6)	4 (57.1)
–Cook (2-3)	2 (12.5)	2 (22.2)	0 (0.0)
–Window cleaner (3)	1 (6.3)	0 (0.0)	1 (14.3)
–Truck driver (3)	1 (6.3)	1 (11.1)	0 (0.0)
–House cleaner (3)	1 (6.3)	0 (0.0)	1 (14.3)
–Employee at bicycle shed (3)	1 (6.3)	1 (11.1)	0 (0.0)
–Building contractor (2)	1 (6.3)	0	1 (14.3)
Working hours per week [median (IQR)]	32 [20.0–36.0]	32 (23.8–26.0)	32 (20.0–36.0)

Data is presented as *n* (%) or *n*, unless otherwise stated. Missing data: working hours per week 1/16, participant in MSC(+) group. ^∞^Tested muscles are shown in appendix 1. ^#^Threshold detection was considered normal if ≥2 out of three touches with Semmes Weinstein monofilament 2.83 were sensed; diminished light touch if <2 touches with monofilament 2.83 were sensed and ≥2 out of three touches with monofilament 3.61 were sensed; diminished protective sensation if <2 touches with monofilament 3.61 were sensed and ≥2 out of three touches with monofilament 4.31 were sensed [39]. If none of the monofilaments were sensed, individuals were classified as no threshold detected. ^*^Dot categories: 1 sedentary work, 2 light work, 3 medium, 4 heavy/very heavy work. *Abbreviations:* BPI, brachial plexus injury; DOT, dictionary of occupational titles, IQR, interquartile range; MRC, Medical Research Council; NA, not applicable; NRS, numeric pain rating scale and SD, standard deviation.

### 3.1 Part 1: Concurrent validity methods for determining physical work demands

All participants declared that they performed the observed tasks regularly on the job. Three participants declared that they also had to perform other tasks during their workday, which were not observed. Table 3 shows an overview of the occupations of the participants and the observed tasks. Strong correlations were observed between the scores obtained for the four observational methods used to determine upper extremity work demands, with the exception of a moderate correlation observed between RULA and Hand activity for TLV (Table 4). The DOT categories were moderately correlated with the OCRA checklist, RULA, and Hand activity for TLV and strongly correlated with the SI. Correlation coefficients between the UEWD-R and other methods were weak to fair.

**Table 3:** Occupations, work place and observed tasks during the work observation

Participant	1 or 2	Occupation	Work place	Description of	Task
	handed*			observed tasks	selected
1	2	Administrative work	Office	Digitalizing documents, using the scanner and computer	Scanning document
2	2	Cook	Professional kitchen	Cutting fish and vegetables	Cutting fish
3	1	Employee at bicycle shed	Semi-covered shed	Labeling bicycles, sweeping the floor, cleaning the ceiling, moving parked bicycles	Sweeping the floor
4	2	Administrative work	Office	Computer work and hands-free phone calls	Computer work
5	2	Building contractor	Construction side and office	Chairing a meeting, supervising construction workers.	Chairing a meeting
6	1	Administrative work	Office	Computer work and hands-free phone calls	Computer work
7	2	Administrative work	Office	Computer work and hands-free phone calls	Computer work
8	1	Administrative work	Office	Putting letters in an envelope, printing documents, computer work.	Putting letters in an envelope
9	1	Administrative work	Office	Computer work	Computer work
10	2	Administrative work	Office	Phone calls using a mobile phone, computer work	Computer work
11	1	Administrative work	Office	Computer work, put documents in a folder	Putting documents in a folder
12	2	Window cleaner	Outdoors	Cleaning windows using a telescopic window cleaner.	Cleaning windows using a telescopic window cleaner
13	2	Truck driver	Outdoors	Driving a truck, loading and unloading of roll containers containing fruits	Unloading of roll containers
14	2	Cook	At home	Cutting vegetables, mixing and crushing vegetables	Mixing and crushing vegetables with both hands
15	1	House cleaner	At home	Dusting and vacuuming	Vacuuming
16	2	Administrative work	Office	Computer work	Computer work

*Work performed one or two-handed.

**Table 4:** Correlations between physical work demand methods scores and risk levels

	UEWD-R	Hand activity for TLV		OCRA checklist		SI		RULA
		Score (*r*)	Risk level (*r*)	Score (*r*)	Risk level (*r*)	Score (*r*)	Risk level (*r*)	Score (*r*)
UEWD-R	–	0.10	–	–	–	–	–	–
OCRA	0.23	0.85*	0.54	–	–	–	–	–
SI	0.23	0.84*	0.42	0.94*	0.91*	–	–
RULA	0.13	0.73*	0.55*	0.85*	0.57*	0.82*	0.55*	–
DOT	0.41	0.79*	–	0.74*	–	0.81*	–	0.65*

Spearman ranked-order correlation coefficients were determined between all physical work demand methods scores and between risk levels (low, medium and high risk) determined with the hand activity for TLV, OCRA checklist, SI and RULA. *Abbreviations:* DOT, Dictionary of Occupational Titles; Hand activity for TLV, Hand activity for Threshold Limit Value; OCRA, Occupational Repetitive Actions; *r*, Spearman’s rho; RULA, Rapid Upper Limb Assessment; SI, Strain Index and UEWD-R, Revised Upper Extremity Work Demands. *p≤0.05.

Risk levels of the OCRA checklist and SI were strongly correlated with each other and fairly correlated with those of the Hand activity for TLV and RULA. Risk levels of the RULA and Hand activity for TLV evidenced fair correlations (Table 4).

### 3.2 Part 2: MSCs, physical work demands, and functional capacity

The median time between performance of the FCE-OH tests and work observation was 32.5 days (interquartile range: 15.5–103.8 days). This time extended beyond 6 weeks for four participants, none of whose DMQ scores changed by more than three points. Therefore, they did not repeat the FCE-OH test. One participant switched jobs, but the type of work and working conditions were similar to those of the previous job. In light of the results obtained in the first part of this study, physical work demands were determined using the OCRA checklist, SI, RULA, Hand activity for TLV, and the DOT methods. The UEWD-R was excluded because of weak to fair correlations with all other measures.

#### 3.2.1 Physical work demand scores and MSCs

The results of the work demand assessments and FCE tests for individuals with and without MSCs are shown in Table 5. Median scores for the OCRA checklist, SI, Hand activity for TLV, RULA, and the DOT were similar for both groups. Assessed risk levels for MSCs across methods (the OCRA checklist, SI, RULA, and Hand activity for TLV) for each individual varied considerably. Notably, they ranged from acceptable to very high levels for five participants. Weak to fair correlations were found between the physical work demand assessment methods and the presence of MSCs (Table 6).

**Table 5:** Work observation and FCE-test results of individuals with BPI with and without MSC

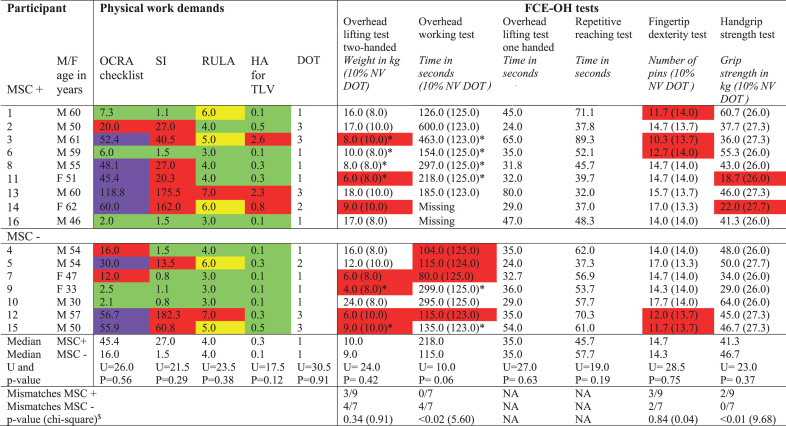

Physical work demands and FCE-OH test results of individuals with BPI with (MSC+) and without MSC (MSC-). Two-handed FCE-OH tests (overhead lifting test two-handed and overhead working test) were performed one-handed if individuals were not able to perform the tests two-handed because of limitations in function of the affected upper limb. The one-handed FCE-OH tests (The overhead lifting test one-handed, repetitive reaching test, fingertip dexterity test and handgrip strength test) were performed with the unaffected upper limb. FCE-OH test results were compared to the tenth percentile of the FCE reference value (10% NV) belonging to the DOT of an individual’s occupation [23]. A match was defined as the FCE-OH test result equals or exceeds the 10% NV, mismatches were marked red. No 10% NV values were available for the overhead lifting test one-handed and the repetitive reaching test. *P*-values (chi-square) represent differences between matches in the MSC- and MSC+group. Physical work demands were measured using the OCRA checklist, SI, RULA, Hand activity for TLV and DOT. *Dot categories:* 1 sedentary work, 2 light work, 3. medium, 4 heavy/very heavy work. *Physical work demands and risk level for the risk on MSC represented by color codes:* OCRA checklist: green, acceptable; yellow, uncertain risk; red, medium risk; purple, high risk. SI: green, probably safe; yellow, increased risk on MSC; red, job is hazardous. RULA: green acceptable; yellow, adjustment is needed in short time; red, immediate improvement is needed. Hand activity for TLV: green, below action limit; yellow, slightly elevated risk; red, significantly elevated risk. *Abbreviations:* DOT, Dictionary of Occupational Titles; F, female; HA for TLV, Hand activity for Threshold Limit Value; kg, kilogram; M, male; MSC, musculoskeletal complaints; NA, not applicable; OCRA, Occupational Repetitive Actions; RULA, Rapid Upper Limb Assessment; SI, Strain Index; U, Mann Whitney U; UEWD, Upper Extremity Work Demands; 10% NV DOT, tenth percentile of the FCE reference value belonging to the DOT of an individual’s occupation. *FCE-OH test performed one-handed. ^$^All chi-square test had one degree of freedom.

**Table 6:** Correlations between FCE-OH test results, physical work demands and MSC

	OCRA	SI	RULA	Hand activity	DOT	MSC
	checklist	Score (*r*)	Score (*r*)	for TLV	Score (*r*)	(*r*)
	Score (*r*)			Score (*r*)
MSC	–0.15	–0.28	–0.23	–0.40	–0.03	–
FCE-OH test
–Overhead lifting test two-handed	–0.20	–0.08	–0.13	–0.04	0.07	–021
–Overhead working test	–0.03	0.20	0.03	0.46	0.17	–0.51
–Overhead lifting test one-handed	0.10	0.16	0.28	0.09	0.17	–0.12
–Repetitive reaching test	–0.23	–0.25	0.04	–0.32	–0.08	0.34
–Fingertip dexterity test	0.19	–0.10	–0.26	0.05	–0.11	0.08
–Handgrip strength test	–0.26	–0.22	0.06	–0.37	–0.08	0.23

*Missing data:* overhead working test 2/16. *Abbreviations:* DOT, Dictionary of Occupational Titles; FCE-OH, functional capacity evaluation one-handed; Hand activity for TLV, Hand activity for Threshold Limit Value; OCRA, MSC, musculoskeletal complaints; Occupational Repetitive Actions; *r*, Spearman’s rho; RULA, Rapid Upper Limb Assessment and SI, Strain Index.

#### 3.2.2 FCE-OH test results and MSCs

The FCE test performances of the MSC- and MSC+groups were similar, with the exception of those relating to the overhead working test, which was performed better by the MSC+group (Table 5). Correlations between FCE-OH test results and the presence of MSCs were weak to fair (Table 6).

#### 3.2.3 Mismatches and MSCs

FCE-OH test results evidenced weak to fair correlations with the physical work demand assessment methods (Table 6). The scatterplots of the overhead lifting tests versus all methods for assessing physical work demands showed that no relations existed among FCE-OH test results, physical work demands, and the presence of MSCs (Appendix 4).

A comparison of the FCE test results and the DOT scores of individuals with BPI with the FCE reference values showed that there were significantly more mismatches for the hand-grip strength test among participants in the MSC+group compared with those in the MSC- group. By contrast, there were significantly fewer mismatches for the overhead working test for the MSC+group (Table 5). Only five participants (four in the MSC+group and one in the MSC- group) showed no mismatches.

## 4 Discussion

Part one of this study aimed to determine the concurrent validity of the physical work demand methods and showed that in, contrast to our hypothesis, the concurrent validity of the physical work demands method methods was only satisfactory for the OCRA checklist and SI. The OCRA checklist scores also showed strong correlations with the Hand activity for TLV and RULA, like the RULA and SI scores. However, the correlation between the risk levels was only fair. In agreement to our hypothesis the DOT was moderately to strongly correlated with the observation-based methods (OCRA checklist, SI, RULA and Hand activity for TLV). The UEWD-R was weakly to fairly correlated with the other work demand assessment methods. In the second part of this study the relationship among MSCs, physical work demands and functional capacity in individuals with BPI was explored In contrast to our hypothesis no relations were observed between the presence of MSCs and physical work demands or between MSCs and functional capacity. It would appear that MSCs are unrelated to mismatches between physical work demands and functional capacity.

### 4.1 Part 1: Concurrent validity methods for determining physical work demands

In this part of the study four observation-based methods were compared together with the DOT and UEWD-R. To the best of our knowledge, not one study has compared all these methods before. Previous studies compared two or three observation-based methods for assessing physical work demands on the upper extremities (SI versus RULA [45], TLV for HAL versus SI and OCRA [46], SI versus OCRA [47], and TLV for HAL versus OCRA [48]). The results of these studies differed because of the use of different methods. Some studies compared the raw scores, while others compared the scores for the risk of MSCs. Similar to our results, those of one study revealed moderate to strong correlations and agreement between the SI and OCRA checklist scores (*r* = 0.94, kappa 0.76) [47]. Another study found that high SI scores were associated with high RULA scores in 75% of the cases [45]. As with our results, studies that compared risk levels for MSCs showed lower levels of agreement between the methods (TLV for HAL versus SI (kappa 0.45) and RULA versus SI (kappa 0.11)) [45, 48]. As in the general working population, the observational methods did not appear to be exchangeable when determining the risk levels of jobs for individuals with BPI [24]. Reassessment of the cutoff points of the risk levels is needed because although the total scores were moderately to strongly correlated, the risk levels were mostly only fairly correlated. The accuracy of the methods may be improved using a video based physical demand description tool [49]. In the absence of demonstrated superiority of one method over the other, the RULA method may be preferred because it is relatively easy to use and applicable to the entire body. However, depending on the aim of the observation and the rater’s experience, another method could also be selected [24].

In light of the results of the first part of this study, the UEWD-R does not seem to be a good screening instrument for work demands on the upper extremities in individuals with BPI. The UEWD-R scores were unrelated to those of the other methods for measuring physical work demands. This finding contrasts with that of a previous study, which showed moderate to good correlations between the UEWD-R scores and RULA C (*r* = 0.69) and RULA D subscores (*r* = 0.65) for the general working population [18]. Individuals with BPI may have more difficulty estimating their physical work demands, resulting in either underestimation or overestimation. Therefore, further research on this topic is recommended.

### 4.2 Part 2: MSCs, physical work demands, and functional capacity

It is striking that 12 out of 16 individuals with BPI had an increased risk for work-related MSCs as assessed with at least one of the observation-based methods. Previous studies conducted on the general working population showed that the relative risk of MSCs increased when individuals had high RULA, TLV for HAL, or SI scores [19–21, 23, 50, 51]. Although our results of the second part of this study showed no direct relation between MSCs and physical work demands, the high risk scores that were observed indicate that working conditions of individuals with BPI need to be evaluated. Adjusting the workplace, the use of assistive devices, or training that aims to improve the performance of the work, may all help to lower physical work demands and to improve the working conditions.

Results of the second part of this study showed that only five (31%) of the participants had sufficient functional capacity to meet their physical work demands. However, our results also showed that MSCs in individuals with BPI appear to be unrelated to a mismatch in physical work demands and functional capacity. The relationship between the presence of MSCs and mismatches has not been previously studied. Our results indicated that as in the general population, MSCs in individuals with BPI are multifactorial [52–54]. Consistent with current views on MSCs, clinicians should therefore not only focus on physical risk factors for MSCs, but should also consider psychological and social factors. Future studies should also incorporate other factors that were not examined in this study but that could have influenced relations between the presence of MSCs and mismatches, such as leisure activities (e.g., playing sports) and recovery time.

### 4.3 Strengths and limitations

A strength of the study was the live work observations that yielded insight into the relations among MSCs, functional capacity, and physical work demands. By observing the participants during their actual work tasks in their own workplace, we succeeded in collecting valuable information on the physical demands required during work. Because of the explorative nature of this study and in order not to miss any relevant results for the development of hypothesis for follow-up research, we did not correct for multiple testing and considered a *p-*value ≤0.1 significant. This may have increased the chance on a type 1 error and the possibility of false positive results. Furthermore, the power of analysis was low, because of the relatively small sample size. Although the sample size was small, it was representative for the BPI population, because most participants were males who acquired BPI because of a trauma at young adult age, which is typical for other BPI populations presented in the literature [1].

In the first part of this study the correlations between physical work demands methods may have been influenced by the low number of occupations analyzed in this study. More than half of the participants performed administrative work, which may have influenced the correlations found between the five methods of assessing upper extremity work demands and the DOT. Therefore, our results may not be generalized directly to other occupations. Correlations may also have been influenced by the fact that observers were not blinded for the scores of the other methods assessing physical work demands.

Results of the second part may have been influenced by the fact that most participants were middle aged, despite we approached potential participants in the age range of 18 to 65 years. We do not expect that age influenced the relationship between MSCs, physical work demands and functional capacity. However, a higher age (>45 years) in the general working population is associated with a decreased functional capacity [55]. Potentially this may have resulted in more mismatches between functional capacity and physical work demands if physical work demands were not adjusted to the possible decreased functional capacity due to aging.

For three participants, not all tasks generally performed during a workday were observed, which may have resulted in the underestimation of the physical work demands of these participants, if the unobserved tasks required higher demands.

In order to define mismatches between FCE-OH test results and physical work demands in the second part of this study, we compared the former to the tenth percentile of the FCE reference values, wherever available [13]. Within the general working population, the tenth percentile of the FCE reference values is considered a valid cutoff point for work that is sedentary or entails light physical demands. For jobs with higher physical demands, there is no valid cutoff point, but the thirtieth percentile seems to be the most appropriate cutoff point [44]. The reference values used for individuals with jobs categorized as DOT 3 may therefore be too low.

Although we showed that the DOT was moderately to strongly correlated with work demands on upper extremities in individuals with BPI, it is not known whether physical work demands represented by the DOT of individuals with BPI are similar to those of the general working population. Physical work demands of individuals with BPI may be higher compared with those of the general working population because the former need to compensate for the loss of function of an upper limb, which may impose an increased load on the unaffected bodily structures. The FCE reference values may therefore be too low for individuals with BPI.

Furthermore, a comparison with FCE reference values may not be appropriate for the overhead working test. Not all participants were able to use both hands to perform the test. Consequently, the test was performed differently by these individuals compared with the performance of the test on which the FCE reference values were based [13]. Individuals with MSCs performed the test one-handedly more often than those without MSCs. It is known that individuals with BPI who performed the test two-handedly performed worse, probably because their affected sides limited the time of their overhead working [25]. This factor may also explain the higher number of mismatches in the MSC- group.

Although we used the “health part 2” of the DMQ to measure MSCs, it does not differentiate between MSCs and neuropathic pain. Therefore, we opted to classify pain in the affected arm as neuropathic pain instead of an MSC. Using this definition of MSCs, we categorized two individuals with pain in the affected side as MSC.

## 5 Conclusion

The first part of this study showed that the concurrent validity of the scores obtained for the OCRA checklist, SI, RULA, and Hand activity for TLV was moderate to good in individuals with BPI. The risk levels for these four observation-based methods were fairly correlated. The DOT category was moderately to strongly correlated with the scores obtained for the observation-based methods. However, differing from our hypothesis, the correlations of the UEWD-R with the other methods was weak to fair. Therefore, it does not appear to be a good screening instrument for work demands on upper extremities in individuals with BPI.

The second part showed that although the observation-based methods indicated that most individuals with BPI had an increased risk of MSCs, our findings suggested that MSCs were not directly related to physical work demands; nor were they related to a mismatch between functional capacity and physical work demands. Moreover, our findings indicate that the cause of MSCs in individuals with BPI, as in the general population, is multifactorial. Clinicians should therefore not only consider physical factors in the treatment of MSCs in individuals with BPI, but also psychosocial factors. Future studies should focus not only on physical work demands but they should also incorporate leisure activities, recovery time, and psychological and socialfactors.

## Ethical approval

This study was approved by the medical ethics committee of the University Medical Center Groningen, The Netherlands (METC 2016.508).

## Informed consent

All participants provided written informed consent before entering the study.

## Conflicts of interest

The authors declare that they have no conflict of interest.

## Supplementary Material

Supplementary Material
