# Supplementary material for: Musculoskeletal complaints, physical work demands, and functional capacity in individuals with a brachial plexus injury: An exploratory study
Source: Work. 2024 Mar 8;77(3):811–25. doi: 10.3233/WOR-220680 (PMC10977377; doi:10.3233/WOR-220680)
Supplement: Supplementary Material [file wor-77-wor220680-s001.docx]

**Appendix 1.** Muscles selected for strength testing

| Selected muscle for testing | Peripheral nerve innervating the muscle |
| --- | --- |
| Trapezius muscle | Accessory nerve |
| Serratus anterior | Dorsal scapular nerve |
| Pectoralis major | Lateral and medial pectoral nerves |
| Supraspinatus | Suprascapular nerve |
| Teres major | Subscapular nerve |
| Deltoid | Axillary nerve |
| Biceps | Musculocutaneous nerve |
| Triceps | Radial nerve |
| Brachioradialis | Radial nerve |
| Pronator teres | Median nerve |
| Supinator | Radial nerve |
| Wrist flexors | Ulnar and median nerve |
| Wrist extensors | Radial nerve |
| Extensor digitorum muscle (2^nd^ digit) | Radial nerve |
| Flexor digitorum superficialis muscle (2^nd^ digit) | Median nerve |
| Opponens pollicis | Median nerve |
| Abductor digiti minimi | Ulnar nerve |
| Adductor pollicis muscle | Ulnar nerve |

**Appendix 2.** Questionnaire additional factors OCRA checklist

OCRA checklist (version 2013) [30]

| I use inadequate gloves more than half of the time | yes/no |
| --- | --- |
| I need to make 2 or more sudden, jerky movements per minute | yes/no |
| I make at least 10 repeated impacts (use hands as tools to hit) per hour | yes/no |
| I work with vibrating tools more than 1/3 of the time | yes/no |
| I use tools that cause blisters, calluses or a red skin | yes/no |
| I need have contact with cold surfaces (less than 0^◦^C) or performance of tasks in cold chambers more than half of the time | yes/no |
| More than half of the time is spent performing precision tasks (on 2 a 3 mm) | yes/no |
| Working pace is set by the machine, but I can speed up or slow down the pace | yes/no |
| Working pace is completely determined by the machine | yes/no |

Which working schedule reflects your working day the best?

| **Description of working schedules** |  |
| --- | --- |
| A short break of 8-10 minutes every hour |  |
| Shift of two hours without a break |  |
| Shift of 4 hours with 1 break and lunch break |  |
| Shift of 4 hours with a lunch break only |  |
| Shift of 7 hours with 2 breaks and a lunch break |  |
| Shift of 8 hours with 2 breaks and a lunch break |  |
| Shift of 7 hours with 1 break and a lunch break |  |
| Shift of 7 hours with a lunch break only |  |
| Shift of 8 hours with 1 break and lunch break |  |
| Shift of 8 hours with a lunch break only |  |
| Shift of 8 hours without breaks |  |
| Other | Please describe: |

**Appendix 3.** Objectives, instructions and outcomes of the functional capacity evaluation One-Handed (FCE-OH) tests.

| **FCE-OH test** | **1 or 2 handed test** | **Objective** | **Test description** | **FCE outcome** |
| --- | --- | --- | --- | --- |
| Overhead lifting test 2-handed | 2 | Strength of shoulder and arm musculature | Lift the plastic receptacle containing weights from table height to crown height 5 times within 90 seconds. After every 5 lifts the weight is increased till the maximum weight in reached. | The maximum lifted weight (kg) |
| Overhead working test | 2 | Static holding time of shoulder and neck musculature | Stand with the hands on crown height with a cuff weight of 1 kg around the wrist of the unaffected side. Manipulate nuts and bolts. Hold this position as long as possible | Time this position is held (sec) |
| Overhead lifting test 1-handed | 1 | Strength of shoulder and arm musculature | Lift a weight of 1.9 Kg 20 times from table height to crown height with one hand | Time needed for 20 lifts (sec) |
| Repetitive reaching test | 1 | Fast repetitive reaching movements of the upper extremity | Sit between two clicking systems on wing span and alternate clicking each button for a total of 30 times as fast as possible | Time needed to press each button 30 times  The mean of 3 trails per arm (sec) |
| Fingertip dexterity test | 1 | Fingertip dexterity | Sit in front of the pegboard (Purdue pegboard, model 32020 J.A. Preston Corporation New York, NY, USA) and place the pins in the board as fast as possible. 3 times per arm, alternating when applicable. | The number of pins placed in the board in 30 seconds  The mean of 3 trails per arm |
| Handgrip strength test | 1 | Grip strength | Patient sitting with shoulder adducted and neutrally rotated, elbow at 90 degrees, forearm and wrist in neutral position. Squeeze a hydraulic hand grip dynamometer (dynamometer, JAMAR, Sammons Preston inc. Bollingbrook, IL, USA) for approximately 3 seconds. 3 times per arm, alternating when applicable. | The mean force of 3 trails per arm |

**Appendix 4.** Scatterplots of FCE-OH test results versus physical work demands


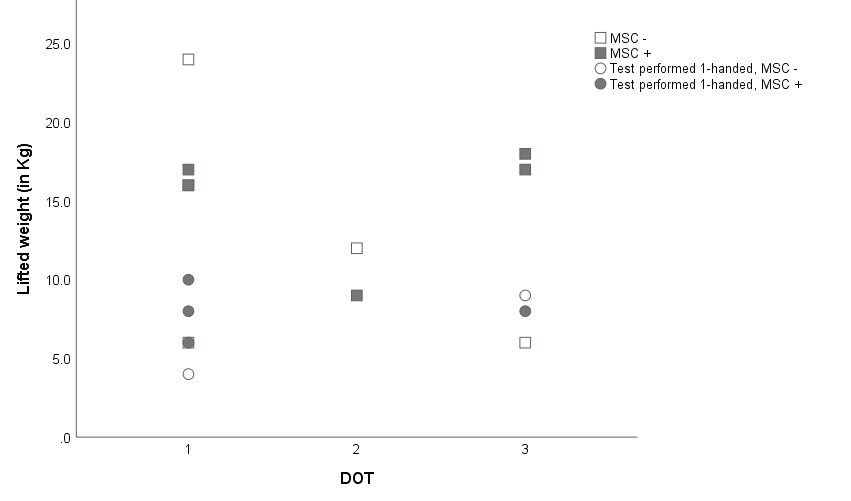

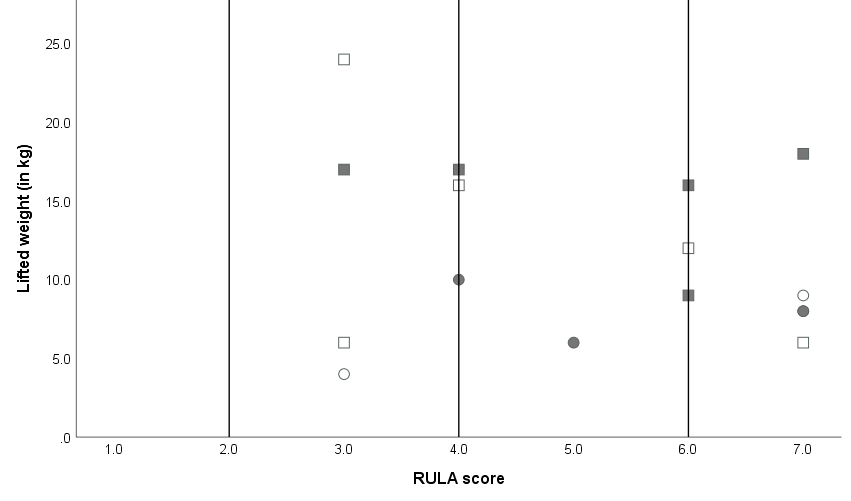


a b


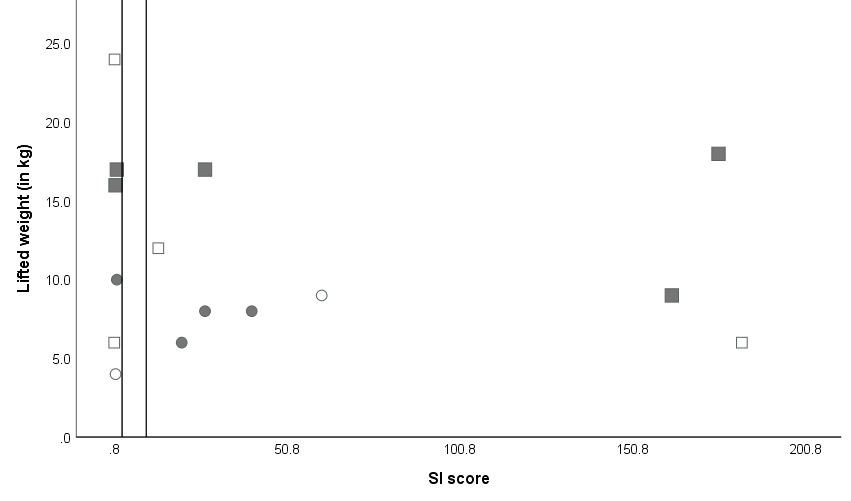


c d

e


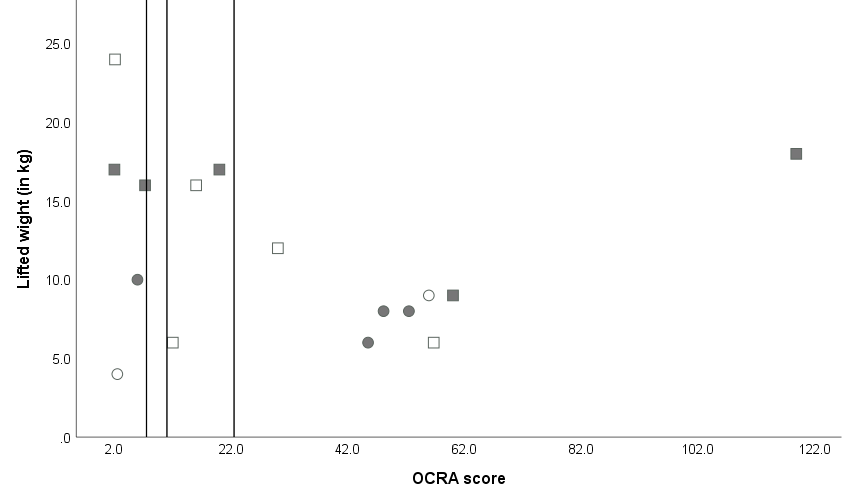

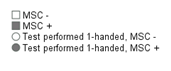

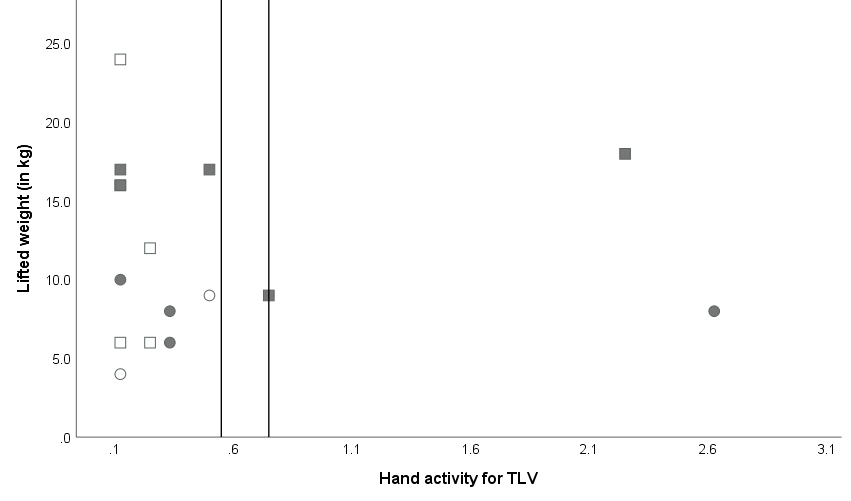


**Figure 1.** Scatterplots of the overhead lifting test two-handed results versus physical work demands of individuals with brachial plexus injury with and without MSC.

Individuals performed the test one-handed if they had too limited remaining activity in the affected limb (○). Individuals with MSC were marked gray and individuals without MSC were marked white.

(a) DOT; 1 sedentary work, 2 work with light physical demands and 3 work with medium physical demands. (b) RULA, the reference lines represent the RULA cut of points (≤2.0 acceptable, 2.0-4.0 further investigations needed, 5-6 adjustments needed, 7 adjustments needed immediately). (c) OCRA checklist, the reference lines represent the OCRA checklist cut of points (≤7.5 acceptable, 7.6-11 limited risk, 11.1-22.5 low-medium risk, > 22.5 high risk). (d) SI, the reference lines represent the SI cut of points (≤3.0 job is probably safe, 3.1-6.9 job may place individual at increased risk for distal upper extremity disorder, ≥7.0 job is probably hazardous). (e) Hand activity for TLV, the reference lines represent the Hand activity for TLV cut of points (< 0.56 green, 0.56-0.78 yellow, >0.78 red).

*Abbreviations:* DOT, Dictionary of Occupational Titles; Hand activity for TLV, Hand activity for Threshold Limited Value; Kg, kilogram; MSC, musculoskeletal complaints; OCRA, Occupational Repetitive Action; RULA, Rapid Upper Limb Assessment and SI, Strain index.


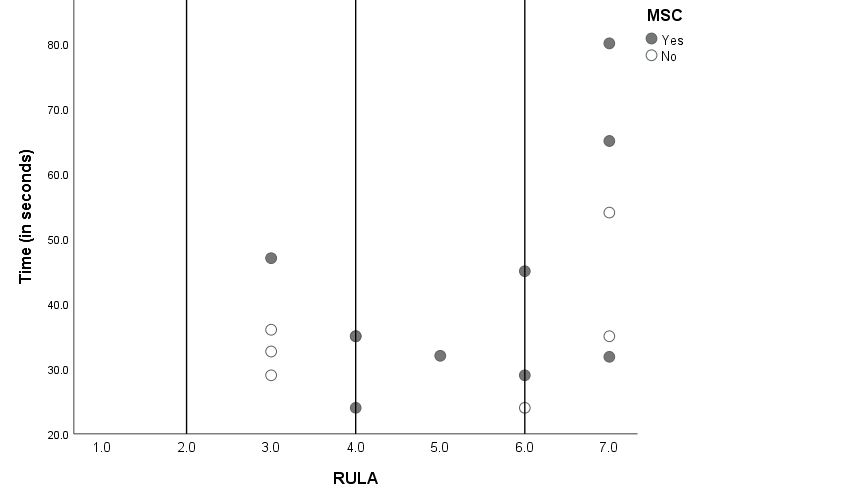

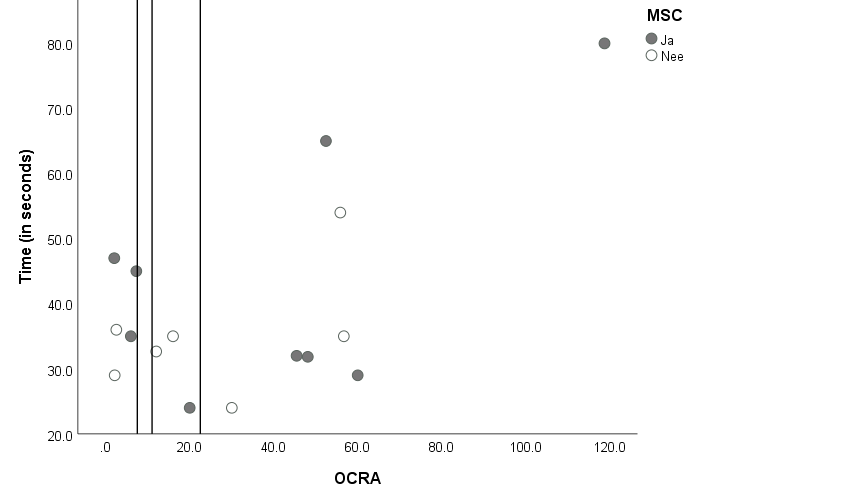

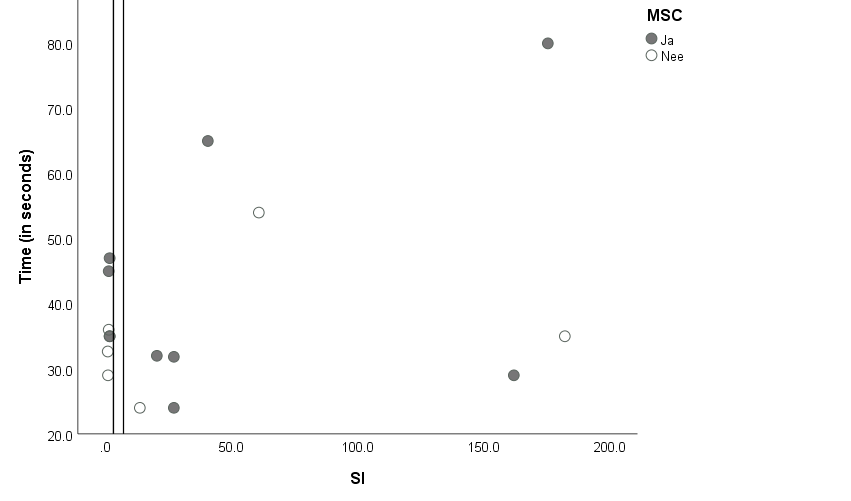


a b

c d

e


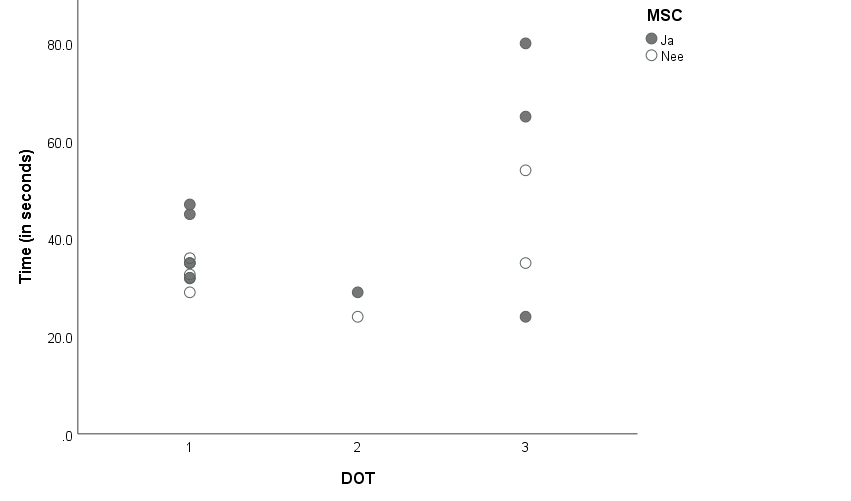

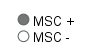

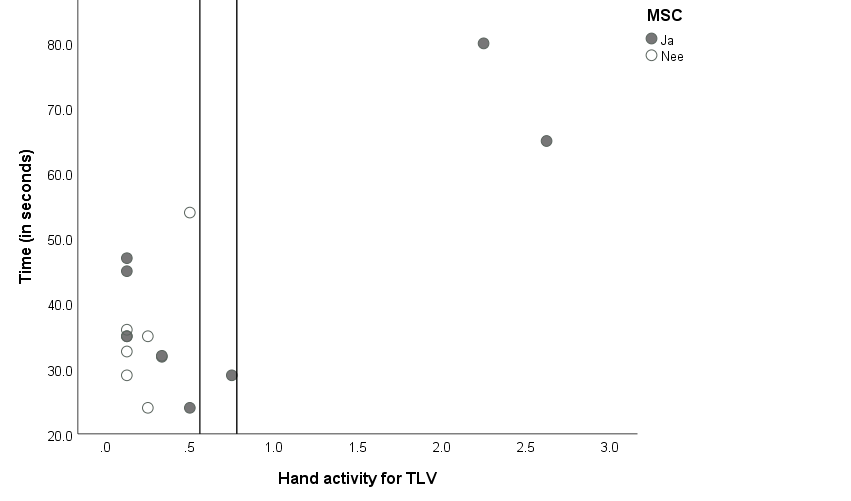


**Figure 2.** Scatterplots of the overhead lifting one-handed test results versus physical work demands of individuals with brachial plexus injury with and without MSC.

Individuals with MSC were marked gray and individuals without MSC were marked white.

(a) DOT; 1 sedentary work, 2 work with light physical demands and 3 work with medium physical demands. (b) RULA, the reference lines represent the RULA cut of points (≤2.0 acceptable, 2.0-4.0 further investigations needed, 5-6 adjustments needed, 7 adjustments needed immediately). (c) OCRA checklist, the reference lines represent the OCRA checklist cut of points (≤7.5 acceptable, 7.6-11 limited risk, 11.1-22.5 low-medium risk, > 22.5 high risk). (d) SI, the reference lines represent the SI cut of points (≤3.0 job is probably safe, 3.1-6.9 job may place individual at increased risk for distal upper extremity disorder, ≥7.0 job is probably hazardous). (e) Hand activity for TLV, the reference lines represent the Hand activity for TLV cut of points (< 0.56 green, 0.56-0.78 yellow, >0.78 red).

*Abbreviations:* DOT, Dictionary of Occupational Titles; Hand activity for TLV, Hand activity for Threshold Limited Value; MSC, musculoskeletal complaints; OCRA, Occupational Repetitive Action; RULA, Rapid Upper Limb Assessment and SI, Strain index.
